# *Suillus indicus* sp. nov. (Boletales, Basidiomycota), a new boletoid fungus from northwestern Himalayas, India

**DOI:** 10.1080/21501203.2014.988770

**Published:** 2014-12-11

**Authors:** Balwant Verma, M. Sudhakara Reddy

**Affiliations:** Department of Biotechnology, Thapar University, Patiala, Punjab147004, India

**Keywords:** *Suillaceae*, ectomycorrhizal, ITS, phylogeny, taxonomy

## Abstract

The new species *Suillus indicus* is described based on the morpho-anatomical description and molecular analysis of basidiomes found in conifer forests of the northwestern Himalayas, India. Morphologically, the key diagnostic characteristics of the new taxon are brownish-orange to reddish-brown pileus with low obtuse umbo, brownish-red to reddish-brown fibrillose squamules over the pileal surface, and absence of fibrillose squamules and glandular dots on the stipe surface. Sequences derived from the internal transcribed spacer region of basidiomes and culture demonstrated that the species is clearly distinct from other known taxa of *Suillus* and new to science.

## Introduction

The genus *Suillus* Gray is one of the most prominent genera of ectomycorrhizal fungi in the order *Boletales* with about 50 species described worldwide (Kirk et al. 2008). *Suillus* species are confined to the temperate, boreal, and Mediterranean regions, although there are few reports (Natarajan & Raman 1983; Halling & Mueller 2002) of their occurrence from tropical areas. Most of *Suillus* species known so far are documented from the northern hemisphere, but some have also been reported from southern hemisphere (McNabb 1968; Watling & Gregory 1989; Dunstan et al. 1998) associated with the introduced pine species. *Suillus* species exhibit narrow host range forming ectomycorrhizae mainly with the members of the family *Pinaceae* and also with some deciduous species (Singer 1986; Kretzer et al. 1996; Wu et al. 2000). Based on the internal transcribed spacer (ITS) sequence analysis, Kretzer et al. (1996) have demonstrated that in the genus *Suillus, Larix* association seems to be primitive and associations with pines, Douglas-fir, and hardwoods seem to be derived in the genus *Suillus*.

The present knowledge of *Suillus* diversity from India, particularly from northwestern Himalayan region, is scanty. *Suillus brevipes* (Peck) Kuntze, *S. pallidiceps* A.H. Sm. and Thiers, *S. punctatipes* (Snell and E.A. Dick) Singer and *S. subluteus* (Peck) Snell have been reported from *Pinus patula* Schltdl. and Cham. forests of Tamilnadu, South India (Natarajan & Raman 1983). *Suillus sibiricus* (Singer) Singer and *S. granulatus* (L.) Roussel have been shown to be ectomycorrhizal with *Pinus wallichiana* A.B. Jacks. (Sagar & Lakhanpal 2005; Dar et al. 2010), whereas *S. triacicularis* Verma and Reddy has been described fruiting exclusively in association with *Pinus roxburghii* Sarg. (Verma & Reddy 2014) from the northwestern Himalayan region of India. In the last few years, we have conducted extensive surveys to the conifer forests of this region with the aim to document and preserve the diversity of *Suillus* and detect some new species of this genus. During our collection trips to northwestern Himalayas, *Suillus* species were found mostly in early monsoon season and *Suillus sibiricus* was observed to be the most frequently encountered and widely distributed *Suillus* species. During our expeditions to Himachal Pradesh in 2010 and 2011, two different specimens of a putative new species showing close morphological resemblance to *S. decipiens* (Peck) Kuntze were collected from mixed conifer forests. In the present study, morphological and molecular data obtained for these specimens are used to clarify the systematic position of the new species *Suillus indicus* within the genus.

## Materials and methods

### Collection sites and fungal isolation

Reference material for the basidiomes has been deposited in the Herbarium of Botany Department (PUN), Punjabi University, Patiala, India, under the voucher numbers PUN 6576 and PUN 6578. Fresh basidiomes of *S. indicus* were collected from Narkanda (latitude 31°27′N and longitude 77°45′E) and Kandyali (latitude 31°13′29″N and longitude 77°26′4″E) sub localities of Shimla, Himachal Pradesh, India. The vegetation of the collection sites is dominated by randomly distributed pure and mixed stands of *P. wallichiana* and *Cedrus deodara* (Roxb. ex D. Don) G. Don.

Malt Extract (ME) agar (2% w/v) media supplemented with streptomycin (50 µg/ml) was used to isolate pure culture. Surface of fresh basidiomes collected was sterilized with rectified spirit and cut along the pileal surface with sterile surgical blades to expose the inner pileal context. Two to three pieces of clean fresh pileal context were transferred to each agar plate and incubated at 25°C for one month. Plates were checked weekly for any contamination and sub-culturing was done, if required. The pure culture isolated for *S. indicus* has been designated as “Isolate SNW02” and submitted to Microbial Type Culture Collection and Gene Bank (MTCC), Institute of Microbial Technology (IMTECH), Chandigarh, India, under the accession number MTCC 11955.

### Morphological description of basidiomes

Standard methodology and terminology used for describing the basidiomes followed Corner (1972). Macro-morphological description, macro-chemical reactions, habitat, and plant association are based on detailed field notes of fresh basidiomes. Color codes in the macroscopic descriptions are from Kornerup and Wanscher (1978). Anatomical features were observed from dried material by reviving the sections either in water or in 3% potassium hydroxide (KOH). Measurements were made at 1000× magnification with a calibrated ocular micrometer on an Olympus light microscope (Olympus, Tokyo, Japan) by mounting the preparations in lactophenol cotton blue. Basidiospores and basidia were measured from the hymenophore of mature basidiomes. The spore measurements exclude the length of apiculus and the basidium length excludes the length of sterigmata. Quotient value (Q = L/W) was calculated considering the mean value of length and width of 20 basidiospores. Microscopic line drawings of microstructures were made from rehydrated material with the aid of a camera lucida.

### Molecular characterization

For phylogenetic analysis of *S. indicus*, genomic DNA from dried basidiome and culture was extracted based on Zhou et al. (1999). DNA extracts were then quantified with a Nanodrop 1000 spectrophotometer (Thermo Scientific, USA) and stored at −20°C until use. The ITS region of nrRNA was amplified with the universal primers ITS1 and ITS4 (White et al. 1990) using a polymerase chain reaction (PCR) Thermal Cycler (Applied Biosystems, Foster City, CA, USA). The thermal cycling conditions applied for the ITS region included an initial denaturation for 5 min at 94°C followed by 34 cycles of 1 min at 94°C, 1 min at 50°C, and 1.5 min at 72°C and a final extension of 7 min at 72°C. PCR products were purified with Qiaquick columns (Qiagen, Hilden, Germany), following the manufacturer’s instructions and sequenced. The ITS sequences obtained from isolate SNW02 and basidiome of *S. indicus* have been deposited in the GenBank under the accession numbers KJ675500 and KJ675502, respectively.

### Phylogenetic analysis

The ITS sequences acquired in this study were compared with those available in the GenBank database by using the BLASTn search algorithm. To find out the possible sister groups for newly sequenced taxon, a preliminary phylogenetic analysis was performed using MrBayes v.3.2.2 (http://mrbayes.sourceforge.net/; Ronquist et al. 2012) by considering the ITS sequences representing all *Suillus* species as mentioned by Bruns et al. (2010). On the basis of preliminary analysis, a group comprising 27 *Suillus* species including our taxon was selected for phylogenetic analysis. Sixty five ITS sequences for other 26 *Suillus* species were retrieved from GenBank. *Suillus triacicularis* (KF977189), which phylogenetically belongs to the *S. granulatus* group (Bruns et al. 2010; Verma & Reddy 2014), was taken as an outgroup taxon. Alignment of the sequences was constructed using MAFFT ver. 7.0 (http://mafft.cbrc.jp/alignment/server/; Katoh & Standley 2013) and edited with BioEdit 5.0.6 (http://www.mbio.ncsu.edu/bioedit/; Hall 1999). The aligned data set has been deposited in TreeBASE (15673). Phylogenetic analysis on the resulting alignment was performed using Bayesian Inference (BI). A Bayesian analysis was implemented in MrBayes v.3.2.2 with two parallel runs each one consisting of four incrementally heated Monte Carlo Markov Chains. The analysis was run using Metropolis-coupled Markov Chains Monte Carlo search algorithm over 2,000,000 generations and the convergence of Bayesian analysis was observed by examination of the standard deviation of split frequencies <0.01. Trees were sampled every 100th generations resulting in total of 20,000 trees. The first 5000 trees, representing the burn-in phase of the analysis, were discarded and the remaining 15,000 trees were used to calculate posterior probabilities (PP) from the 50% majority rule consensus trees.

## Results

### Phylogenetic inference

**Figure 1.**
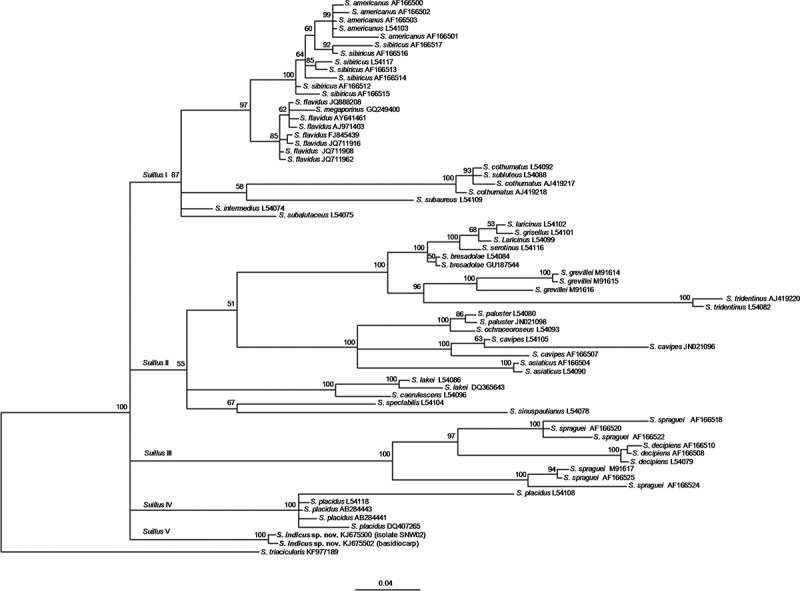
Bayesian tree showing the relationships between the internal transcribed spacer (ITS) sequences derived from the basidiome and isolate SNW02 (indicated in the brackets) of *Suillus indicus* (shown in bold) and those of related species retrieved from GenBank. Numbers at nodes stand for the posterior probability percentages (>50%) of the Bayesian analysis (outgroup: *Suillus triacicularis*).

The PCR products amplified with ITS1 and ITS4 were 702 bp in length. Sequence analysis by BLAST revealed 93% similarity (query coverage of 99%) with unidentified *Suillus* sp. K91S8 (GQ267488) from New Zealand (Walbert et al. 2010) and 92% similarity (99% query coverage) with *S. flavidus* isolate FFP962 (JQ711908) from Canada (Jones et al. 2012). The alignment of ITS sequences of selected *Suillus* species resulted in a data matrix comprising 28 taxa and 713 characters including gaps. Bayesian analysis of ITS region for the selected *Suillus* group yielded a consensus tree (Figure 1) and divided the species broadly into five major clades (*Suillus*I, *Suillus*II, *Suillus*III, *Suillus*IV, and *Suillus*V). The clade *Suillus*V consisted of two ITS sequences of the present study (*S. indicus*) and forms an independent clade in the group, which is well supported by the Bayesian posterior probability percentage (100%). The ITS sequences of *Suillus spraguei* (Berk. and Curt.) Kuntze derived from American (M91617, AF166524, and AF166525) and Chinese collections (AF166518, AF166520, and AF166522) are paraphyletic and sub-divided into two different subclades. Chinese *S. spraguei* isolates were found to be sister to American *S. decipiens* isolates (L54079, AF166508, and AF166510) rather than American *S. spraguei* isolates. Contrastingly, a few pairs of *Suillus* species (*S. flavidus* and *S. megaporinus, S. cothurnatus* and *S. subluteus, S. laricinus* and *S. grisellus*) are not distinguished by the ITS locus.

## Taxonomy

**Figure 2.**
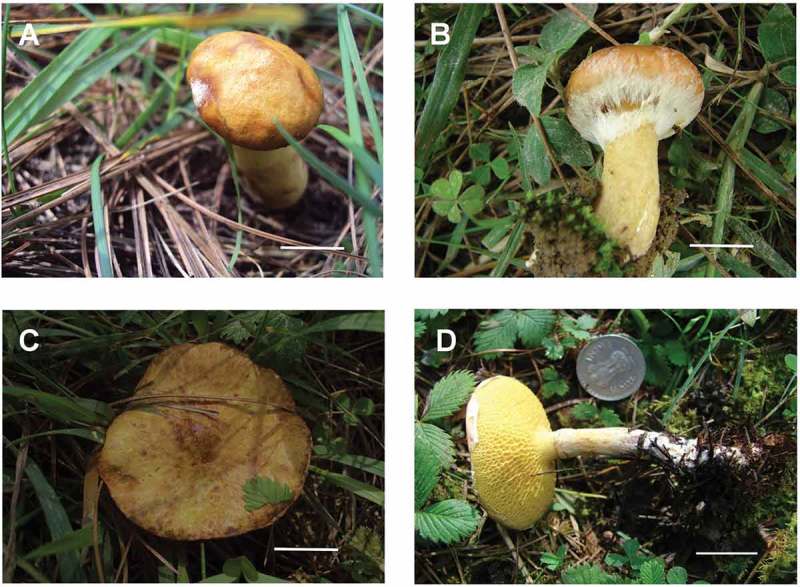
*Suillus indicus* basidiomes: A. Young basidiome showing umbo and very few appressed fibrillose squamules on the pileus; B. Young basidiome showing white partial veil and absence of glandular dots on the stipe surface; C. Mature basidiome with appressed fibrillose squamules and a low obtuse umbo on pileal surface; D. Stipe with annulus and no glandular dots/smears. Scale bars: A–B = 1 cm, C–D = 2 cm.

**Figure 3.**
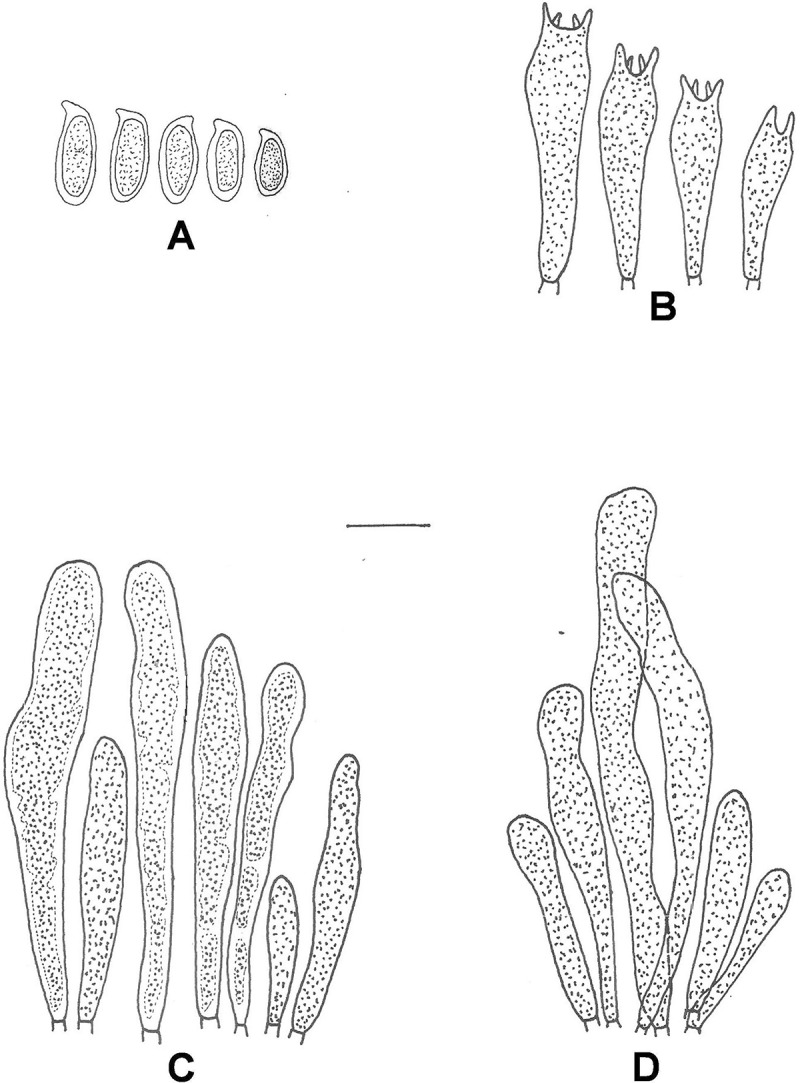
Microscopic line drawings of *Suillus indicus* (holotype): A. Basidiospores; B. Basidia; C. Pleurocystidia; D. Cheilocystidia. Scale bar: A–D = 10 µm.

***Suillus indicus*** B. Verma and M.S. Reddy, **sp. nov.** (Figures 2 and 3). MycoBank No.: MB 808527.

***Basidiomes***: ranges 4.0–10.0 cm in height. ***Pileus***: 3.0–9.0 cm broad, convex when young, flattened or slightly upturned with age; margin regular, appendiculate with white velar fragments (1A1); pileus surface dry to moist, not viscid or slightly viscid only when wet, brownish-orange (6C4–6C6) when young, reddish-brown (8D7–8D8) in age, ground color discoloring to pale yellow with age, low obtuse umbo present and cuticle fully peeling; brownish-red (8C7–8C8) to reddish-brown (8D7–8D8) fibrillose squamules present throughout the pileal surface. Pileus context ≤20 mm thick, soft, pale yellow (2A3) to yellowish (2A6) in age, unchanging or slowly staining pinkish when exposed; taste mild and odor not distinctive. ***Tubes***: up to 8 mm deep, decurrent, radially arranged, fully peeling, yellow (3A6–3A7) when young, brownish-yellow (5C7–5C8) with age, slowly turning light brown on bruising; pore mouths irregular, 1–3 mm broad. ***Stipe***: central, 3.0–8.0 cm long, 1.0–1.2 cm thick, cylindrical and slightly enlarged downward, typically clavate in young specimens, glandular dots absent, concolorous with the tubes but white (1A1) at base; white colored annulus present turning brown with time; basal mycelium at base white. Stipe context solid, pale yellow (2A3–2A6) and turning dirty brown when exposed. ***Basidiospores***: cinnamon brown (6D6) in mass, hyaline to pale yellow in KOH, yellowish in Melzer’s reagent, smooth with granular content inside, 7.5–11.5 × 3.0–4.5 µm in size and cylindric to subcylindric in shape (Q = 2.6). ***Basidia***: 17.0–30.0 × 4.5–6.5 µm, clavate, 4-spored, occasionally 2-spored, hyaline to yellowish in KOH, yellowish with yellowish brown contents in Melzer’s reagent; sterigmata 1.5–3.1 µm high. ***Pleurocystidia***: 18.0–54.0 × 4.0–8.5 µm, cylindrical to subclavate with blunt to constricted tips, scattered or in fascicles, mostly with brown coagulated granular contents inside, hyaline in KOH, yellowish brown in Melzer’s reagent. ***Cheilocystidia***: 20.0–62.0 × 3.0–5.0 µm, shape similar to pleurocystidia, granular, slightly yellowish in KOH, brownish in Melzer’s reagent, abundant and mostly in fascicles. ***Caulocystidia***: absent. Trama divergent. Clamp connection absent.

### 

#### Chemical color reactions

Pileal context: 2.5% KOH – pink to bluish gray, 10% FeSO_4_ – olive gray, 14% ammonia – pinkish red to bluish gray, concentrated HNO_3_ – no color reaction. Pileus cuticle: 2.5% KOH – greenish-black, 10% FeSO_4_ – blue-black, 14% ammonia – dark green to blue-black, concentrated HNO_3_ – no color reaction. Baroni (1978) studied the chemical spot tests of 6 genera of *Boletaceae* and found that pink to reddish color reaction of pileal context with KOH and ammonia distinguishes *Suillus* species from other *Boletaceae*.

#### Habitat

Solitary on humose soil under *Cedrus deodara* trees in mixed forest of *Pinus wallichiana* and *C. deodara*.

#### Specimens examined

INDIA, Himachal Pradesh, Shimla: Narkanda, 2621 m, 27 July 2010, growing solitary on humicolous soil in mixed forest of *P. wallichiana* and *C. deodara, B. Verma* (Holotype: PUN 6576); Kandyali, 2450 m, 29 July 2011, growing solitary on soil in mixed forest of *P. wallichiana* and *C. deodara, B. Verma* (Paratype: PUN 6578).

#### Commentary

The species is morphologically close to *Suillus decipiens* (Peck) Kuntze, but can be distinguished mainly by the absence of fibrillose squamules over the stipe, umbonate pileus and less numerous fibrillose squamules over the pileal surface. Microscopically, the species differs from *S. decipiens* by the presence of few 2-spored basidia on hymenium and absence of caulocystidia on stipe surface.

#### Etymology

The specific epithet *indicus* is taken from Latin with reference to the new species being reported from the Indian region.

## Discussion

In the Bayesian analysis of the *Suillus* group, selected for the phylogenetic inference in the present study, a few pairs of *Suillus* species (*S. flavidus* and *S. megaporinus, S. cothurnatus* and *S. subluteus, S. laricinus* and *S. grisellus*) are not distinguished by the ITS locus suggesting that there are limitations of using ITS locus for species-level identifications in the genus *Suillus*. This might be due to over-description (=synonymy) or the lack of ITS divergence between the sibling species (Bruns et al. 2010; Verma & Reddy 2014). The *S. spraguei* isolates (American and Chinese) analyzed showed remarkable heterogeneity and were divided into two different subclades. Chinese *S. spraguei* clade is sister to *S. decipiens*, whereas American *S. spraguei* is paraphyletic. On the basis of ITS data, Wu et al. (2000) also observed similar heterogeneity among the Chinese and American *S. spraguei* isolates.

The phylogenetic analysis of selected *Suillus* taxa clustered them mainly into five different clades (Figure 1). According to Smith and Thiers (1964) and Klofac (2013), these clades can be morphologically distinguished on the basis of few basic morphological characteristics such as presence or absence of veil, annulus, and glandular dots on the basidiomes. All of the clades, except *Suillus*IV, mainly comprise species exhibiting a well-developed veil or a false veil in young specimens either leaving an annular zone or a true annulus on the stipe or otherwise adhered to the pileal margin at maturity. On the other hand, the clade *Suillus*IV consists of a single species *S. placidus* (Bonord.) Singer, which has no veil and annulus at any stage of the development but glandular dots or smears are present on the stipe (Singer 1945; Smith & Thiers 1964). The first four clades comprising *Suillus* species with a well-developed veil or false veil in young specimens can be further divided on the basis of presence or absence of glandular dots on the stipe. The clade *Suillus*I mainly comprises species with glandular dots, while the clades *Suillus*II, *Suillus*III, and *Suillus*V comprise only species that lack glandular dots on the stipe. Furthermore, the species in clades *Suillus*II, *Suillus*III, and *Suillus*V can be classified on the basis of plant host to which they are found to be associated in nature. All the *Suillus* species in clade *Suillus*II are generally associated either with *Larix, Pseudotsuga, Abies,* or *Picea* (Smith & Thiers 1964; Klofac 2013) as compared to the species in clade *Suillus*III (Kuntze 1898; Smith & Thiers 1964; Wu et al. 2000; Burchhardt et al. 2011; Klofac 2013), which are found to be associated only with *Pinus* species. *Suillus indicus* specimens (clade *Suillus*V) were collected from mixed forests of *P. wallichiana* and *C. deodara* under *C. deodara* trees suggesting its probable association with *C. deodara*, although its association with *P. wallichiana* cannot be denied. In fact, host shifts of basidiomycetes are considered to be major driving forces in the evolution process (Refrégier et al. 2008; Li et al. 2009, 2011; Rochet et al. 2011). Thus, the *Suillus* species of selected group are phylogenetically grouped on the basis of their basic morphological features and the host specificity. The Bayesian analysis clustered *S. indicus* as a distinct clade (*Suillus*V) in the group distinguishing it from all other *Suillus* species.

Morphologically, *S. indicus* come close to *S. decipiens* but differs considerably identifying it as a distinct species. The presence of umbo, less numerous/prominent fibrillose squamules over the pileal surface and absence of squamules over the stipe differentiate it from *S. decipiens*. Anatomically, the occasional presence of two-spored basidia and absence of caulocystidia distinguish the species from *S. decipiens. Suillus spraguei* is the next closest species, which is commonly referred to as *Suillus pictus* A.H. Sm. and Thiers although the name *S. spraguei* is used for one or even several disjunct populations of *S. pictus* in Asia (Wu et al. 2000; Burchhardt et al. 2011; Klofac 2013). The pileus color of young and fresh *S. spraguei* specimens is much redder than *S. decipiens* and *S. indicus* but the faded specimens strongly resemble *S. decipiens*. Furthermore, in contrast to *S. indicus, S. spraguei* also bears fibrillose squamules on the stipe. The combination of morpho-anatomical features and phylogenetic analysis of ITS sequences derived from fruiting bodies and culture distinguishes *S. indicus* as a distinct species and we herein report it as new to science.
